# Leaf Intracellular Water Transport Rate Based on Physiological Impedance: A Possible Role of Leaf Internal Retained Water in Photosynthesis and Growth of Tomatoes

**DOI:** 10.3389/fpls.2022.845628

**Published:** 2022-04-01

**Authors:** Deke Xing, Renlong Mao, Zhenyi Li, Yanyou Wu, Xiaojie Qin, Weiguo Fu

**Affiliations:** ^1^Key Laboratory of Modern Agricultural Equipment and Technology, Ministry of Education, Institute of Agricultural Engineering, Jiangsu University, Zhenjiang, China; ^2^State Key Laboratory of Environmental Geochemistry, Institute of Geochemistry, Chinese Academy of Sciences, Guiyang, China

**Keywords:** electrophysiology, growth, photosynthesis, water potential, water-use efficiency

## Abstract

Water consumed by photosynthesis and growth rather than transpiration accounts for only 1–3% of the water absorbed by roots. Leaf intracellular water transport rate (LIWTR) based on physiological impedance (Z) provides information on the transport traits of the leaf internal retained water, which helps determine the intracellular water status. *Solanum lycopersicum* plants were subjected to five different levels of relative soil water content (SWC_*R*_) (e.g., 100, 90, 80, 70, and 60%) for 3 months. The leaf water potential (Ψ_L_), Z, photosynthesis, growth, and water-use efficiency (WUE) were determined. A coupling model between gripping force and physiological impedance was established according to the Nernst equation, and the inherent LIWTR (LIWTR_*i*_) was determined. The results showed that LIWTR_*i*_ together with Ψ_*L*_ altered the intracellular water status as water supply changed. When SWC_*R*_ was 100, 90, and 80%, stomatal closure reduced the transpiration and decreased the water transport within leaves. Net photosynthetic rate (*P*_N_) was inhibited by the decreased stomatal conductance (g_*s*_) or Ψ_*L*_, but constant transport of the intracellular water was conducive to plant growth or dry matter accumulation. Remarkably, increased LIWTR_*i*_ helped to improve the delivery and WUE of the retained leaf internal water, which maintained *P*_*N*_ and improved the WUE at 70% but could not keep the plant growth and yields at 70 and 60% due to the further decrease of water supply and Ψ_*L*_. The increased transport rate of leaf intracellular water helped plants efficiently use intracellular water and maintain growth or photosynthesis, therefore, adapting to the decreasing water supply. The results demonstrate that the importance of transport of the leaf intracellular water in plant responses to water deficit by using electrophysiological parameters. However, the LIWTR in this research is not directly linked to the regulation of photosynthesis and growth, and the establishment of the direct relationship between leaf internal retained water and photosynthesis and growth needs further research.

## Introduction

Water plays a crucial role in the life of the plant. Most (∼97%) of the water absorbed by a plant’s roots is carried through the plant and transpired from leaf surfaces. Only a small amount of water absorbed by the roots is retained in the plant to supply growth (∼2%) or to be consumed in biochemical reactions (∼1%) (Taiz et al., 2015). Water moves in the plant *via* the apoplast, symplast, and transmembrane pathways, and it faces resistance as it enters and moves through the leaf, which is also called hydraulic conductance (Liu et al., 2014). Leaf is the main site for plant photosynthesis and transpiration (Wright et al., 2004). Water is pulled from the xylem into the cell walls of the mesophyll, where it evaporates into the air spaces within the leaf. Water vapor then diffuses through the leaf air space, through the stomatal pore, and across the boundary layer of still air found next to the leaf surface (Taiz et al., 2015). Even slight imbalances between the uptake and transport of water and the loss of water to the atmosphere can cause water deficits (Scharwies and Dinneny, 2019). In the leaf transport network, the leaf capacitance which is the ability of the leaf to store water and release water into the transpiration stream is thought to be crucial for the maintenance of leaf water balance (Vitali et al., 2016). Plants can also improve their water-holding capacity to cope with water deficit environments (Bucci et al., 2019). However, the transport and availability of the leaf internal retained water (1–3%), especially the intracellular water, are more closely intertwined with the mineral nutrition, water regulation, and photosynthesis compared with the whole absorbed water by roots (Hsiao and Xu, 2000).

A common response in plants to water deficit is stomatal closure to decrease transpiration and limit water loss (Vaziriyeganeh et al., 2018). The instant water-use efficiency (WUE_*i*_) means the amount of carbon fixed in photosynthesis per unit of water transpired. Higher WUE_*i*_ values have been observed in plants with lower stomatal conductance, but these gains are usually achieved together with a reduction in net photosynthetic rate (*P*_*N*_) (Lawson and Blatt, 2014). WUE_*i*_ can be timely measured but the measurements of WUE_*i*_ just investigate the status of the whole absorbed water in plants, and it cannot timely reflect the dynamic variation and utilization of the leaf internal retained water. Water deficit can also lead to a decline in leaf water potential (Ψ_*L*_) and even intracellular water availability, which is a direct cause of the disturbance of essential physiological functions and photosynthetic processes and, therefore, affects plant growth and development (Rodrigues et al., 2020). Some plants can regulate their intracellular water availability to maintain photosynthesis by water regulation-related enzymes (Hu et al., 2011; Lovelli et al., 2019). The decrease in Ψ_*L*_ can be mitigated for a short time due to water regulation caused by water regulation-related enzymes. However, the abovementioned water regulation process has a certain hysteresis. Besides, Ψ_*L*_ mainly governs the transport of water across plasma membranes (Johansson et al., 1998; Taiz et al., 2015), and it can reflect the water status at a certain time point but is difficult to determine the dynamic characteristics of the intracellular water. Some studies have reported that a decline in leaf hydraulic conductance could trigger the decrease of stomatal conductance in rice (*Oryza sativa*) (Flexas et al., 2018). Results in some studies also indicated that the correlation of leaf hydraulic conductance and stomatal conductance may be species-dependent (Xiong et al., 2017). Leaf hydraulic conductance can influence the transport of substances within the leaf and can determine the photosynthesis and its dynamics under shifting environmental conditions, but it is determined as the ratio of the water flow rate through the leaf to the water potential gradient driving force for water movement across the leaf (Flexas et al., 2013). The abovementioned driving force is mainly provided by the transpiration from the leaf surface. At present, there are few reports about the dynamic characteristics of the leaf internal retained water, especially the intracellular water and its related photosynthesis and growth in plants. Studies on leaf intracellular water transport and utilization traits are of great significance to the investigation of the roles of leaf internal retained water in photosynthesis and growth. Determination of leaf intracellular water transport traits together with Ψ_*L*_ will provide detailed information on the status of the leaf internal retained water.

Electrophysiological properties have increasingly been used for diagnosing plant health and water status (Zhang et al., 2015; Najdenovska et al., 2021). The electrophysiological behavior of a plant is closely related to that of a single cell, and the cell can be presumed to be a spherical capacitor. Electrical characteristics vary between the organelles, vacuole, and cytoplasm, which occupy most of the space in cells and can be regarded as resistors, while plasma membrane has capacitive characteristics (Zhang et al., 2020). Electric current is always affected by the resistors, capacitors, and inductors in the alternating current circuit, and impedance is the sum of the resistance to current caused by the resistors, capacitors, and inductors (Schönleber and Ivers-Tiffée, 2015). An electric potential difference is produced when current passes through the cell membrane, and it is retained by the efficient transport system and the alternative permeability of the cell membrane (Lindén et al., 2016). Leaf physiological impedance (Z) is generated by the transport of intracellular dielectric materials, including inorganic and organic ions. Its values change with changing cell water content and cell membrane permeability. Therefore, leaf intracellular water transport traits are correlated with the cell impedance characteristics, which can be rapidly determined by using a nondestructive custom-made parallel-plate capacitor (Xing et al., 2021).

Tomato (*Solanum lycopersicum* L.) is a member of the family *Solanaceae*, which comprises short-lived herbaceous plants. Tomato is a healthy food that is low fat and cholesterol-free and a good source of fiber and protein (Kyari et al., 2020). Tomatoes have a high level of acceptability by people in daily life activities in China (Chen et al., 2020). However, it is also a highly water-demanding crop (Lu et al., 2019), thus requiring irrigation throughout the growing season. Agricultural water consumption accounts for approximately 68.7% of the total water uses in China (Huang et al., 2012). In light of the socioeconomic pressures on the country’s water resources, water demand management in the agricultural sector that leads to greater efficiency in the use of water is of increasing importance. Therefore, research on the roles of leaf internal retained water in photosynthesis and growth also has the potential to accurately determine the water requirement information and improve the WUE of tomatoes.

Here, we aimed to determine the leaf intracellular water transport rate (LIWTR) based on the physiological impedance of tomatoes under different water supplies to provide a basis for further research on the timely monitoring of plant water requirement information. Based on the understanding of leaf intracellular water transport and utilization traits, the roles of leaf internal retained water in photosynthesis, growth, yield, and WUE could be investigated. In this article, tomato seedlings were grown and subjected to different water supplies to study the responses of Z, Ψ_*L*_, chlorophyll contents, photosynthetic characteristics, nutrient contents, growth indices, and WUE. We hypothesized that plants would adapt to decreasing water supply by improving the transport rate and WUE of the leaf intracellular water to maintain the photosynthesis and growth. However, in fact, the direct link between LIWTR and the regulation of photosynthesis and growth cannot be established in this research.

## Materials and Methods

### Plant Growth and Treatment

The research was conducted in a greenhouse at the Institute of Agricultural Engineering, Jiangsu University, Jiangsu Province, China (N 32°11′ and E 119°27′). Tomato (*S. lycopersicum* L.) is the most popular vegetable in the world, and it is also a short-lived and highly water-demanding crop. Research on the timely determination of the water status and water requirement information of this type of crop helps improve the WUE of the agricultural freshwater resources. The cultivar of the tomato used in this experiment was Cooperation 906, which was bred by the Institute of Northern Agriculture and Science in China, and it was characterized by high yield and good stress resistance. Therefore, this variety of tomato plants was selected as experimental materials for the study. The Cooperation 906 tomato seeds were obtained from an online shopping platform in China. Tomato seeds were surface-sterilized with 1% sodium hypochlorite, and then they were germinated and grown for 45 days in several 72-hole trays with organic soil. Later, 50 seedlings uniform in size from those trays were selected, only seedlings with intact roots were used and transplanted into the pots layered with clay, and those transplanted seedlings were grown in pots for another 1 week before the different water supply treatments (just one seedling was grown in each pot). The size of each pot was 19.70 cm in depth, 29.60 cm in top diameter, and 17.80 in bottom diameter, and seedlings were watered daily during the germination and cultivation periods. The greenhouse was in a subtropical monsoon climate zone, with a day/night temperature cycle of 25/17°C and relative humidity of 68 ± 4% during the germination, cultivation, and treatment periods.

Five different water supply treatments were set up in the experiment by subjecting those 50 selected seedlings grown in the pots to five different levels of relative soil water content (SWC_*R*_). SWC_*R*_ of the five treatments was 100% (the control), 90, 80, 70, and 60%, respectively, and the percentage of the field capacity for 100% (the control) was 35.40%. The experiment was arranged in a completely randomized design, every 10 healthy and uniform seedlings were used under each water supply treatment, which meant each treatment had 10 repetitions, and the treatment lasted for 3 months. Water was supplied every day at dusk, and the weight of each pot that contained a plant at each treatment level every day during the whole treatment period was maintained the same as that at the beginning of the corresponding treatment level (SWC_*R*_ at each treatment level would change as time increased compared with that at the beginning because of the growth of plant). The water supply volume at each treatment level was recorded every day. The water consumption per plant at each treatment level during the whole treatment period was then calculated. Of course, measuring soil water potential was more conducive to maintain the soil water status consistent compared with SWC_*R*_, but it would be convenient to record the daily water supply and calculate the water consumption by means of weighing. We conducted the water supply for each treatment at the same time every day to furthest maintain the difference of SWC_*R*_ between different treatments, and the stimulation of different water supplies (e.g., excessive and relatively less water supply) on tomatoes would cause different effects on the water uptake by roots and those physiological and growth indices. As a result, we could successfully analyze the relationship between leaf intracellular water transport traits and photosynthesis and growth. Measurements of plant height, stem diameter, and leaf area were conducted every 12th day after the onset of the treatment. The photosynthesis, electrophysiology, and Ψ_*L*_ parameters were determined at day 90 from the onset of the treatment. The fourth and fifth youngest fully expanded leaves from the top (five plants from each treatment group, about four or five fruits were left in each tomato plant) were chosen for measurements. Samples and data were collected and determined from five plants that are randomly selected from the 10 repetitions at each treatment level.

### Determination of Leaf Water Potential, Water Content, and Leaf Intracellular Water Transport Rate

The variation in Z with increasing gripping forces was measured using an LCR tester (*Model 3532-50*, Hioki, Nagano, Japan), and the frequency and voltage used were 3 kHz and 1 V, respectively. Each leaf was clipped onto the custom-made parallel-plate capacitor (Xing et al., 2021). With a dew point microvoltmeter in a universal sample room (*C-52-SF*, *Psypro*, Wescor, Logan, UT, United States), Ψ_*L*_ was measured at the same position of the leaves with the above Z testing. The leaves were dried in an oven at 80°C, leaf fresh weights (FW_*L*_) and dry weights (DW_*L*_) were determined using an electronic analytical balance (BSA124S, Sartorius, Gottingen, Germany), and leaf water content (LWC, %) was calculated as follows: LWC=(*FW*_L_-DW_L_)/FW_L_.

The following equation was used to calculate gripping forces (F_*g*_, N), which were used for clamping a leaf during the CP and Z measurements:


(1) $$F_{\text{g}}=\left(M_{i}+m\right)\,\text{g}$$

where *F*_*g*_ is gravity (gripping force, N); *M*_*i*_ is the mass of iron (kg); *m* is the mass of the foam board and electrode (kg); and g is the acceleration of gravity with a value of 9.80 N kg^–1^.

The electrophysiological behavior of a plant is closely related to that of a single cell, and the cell can be presumed to be a spherical capacitor. Cell impedance mainly depends on the ratio of ion concentrations between the intramembrane and extramembrane space when the measurement is conducted within a single object in the same situation. Therefore, leaf impedance values change with changing cell water content and cell membrane permeability. The latter can be influenced by the external stimulus, which changes the ion concentrations inside and outside of the membrane. The Nernst equation can be applied to the difference in the ion concentrations mentioned above, and impedance is inversely proportional to intracellular ion concentration at a given extracellular ion concentration. As a result, the relationship between impedance and external stimuli can be derived.

Changes in the permeability of the cell membrane differ between plants at a given gripping force, and the impedance differs between different plants (Xing et al., 2021).

The Nernst equation (Brosmer and Peters, 2012) is as follows:


(2) $$\text{E}-{\text{E}}^{0}=\frac{\text{RT}}{{\text{nF}}_{0}}\,\text{ln}\,\frac{{\text{C}}_{\text{i}}}{{\text{C}}_{\text{o}}}$$

where E is the electromotive force (V); E^0^ is the standard electromotive force (V); R is the gas constant (8.31 J K^–1^ mol^–1^); T is the thermodynamic temperature (K); C_*i*_ is the intracellular ion concentration (mol L^–1^); C_*o*_ is the extracellular ion concentration (mol L^–1^); F_0_ is the Faraday constant (9.65 × 10^4^ C mol^–1^); and n is the ion transfer amount (mol).

The work produced is due to pressure, which is transformed from the internal energy of the electromotive force, and it displays a positive correlation with PV (PV=aE) (Zhang et al., 2021a). Thus,


(3) $$\text{PV}=\text{aE}={\text{aE}}^{0}+\frac{\text{aRT}}{{\text{nF}}_{0}}\,\text{ln}\,\frac{{\text{C}}_{\text{i}}}{{\text{C}}_{\text{o}}}$$

where P is the pressure imposed on leaf cells, Pa; “a” is the transfer coefficient from electromotive force to energy; V is the cell volume, m^3^; P is calculated as $\text{P}=\frac{{\text{F}}_{\text{g}}}{\text{S}}$, where F_*g*_ is the gripping force; and S is the effective area of the leaf that is in contact with capacitor plants, m^2^.

The vacuole and cytoplasm occupy most of the space in the developed mesophyll cells. In terms of the mesophyll cell, the sum of C_*o*_ and C_*i*_ is constant, which is equal to the total ion concentration inside and outside of the membrane. C_*i*_ is positively correlated with electrical conductivity, and the electrical conductivity is the reciprocal of Z (Zhang et al., 2021b). Therefore, $\frac{{\text{C}}_{\text{i}}}{{\text{C}}_{\text{o}}}$ can be expressed as $\frac{{\text{C}}_{\text{i}}}{{\text{C}}_{\text{o}}}=\frac{\frac{\text{f}}{\text{Z}}}{\text{C}-\frac{\text{f}}{\text{Z}}}=\frac{\text{f}}{\text{CZ}-\text{f}}$, where f is the transfer coefficient between C_*i*_ and Z. Equation (3) can be rewritten as follows:


(4) $$\frac{\text{V}}{\text{S}}\,{\text{F}}_{\text{g}}={\text{aE}}^{0}-\frac{\text{aRT}}{{\text{nF}}_{0}}\,\text{ln}\,\frac{\text{CZ}-\text{f}}{\text{f}}$$

Then


(5) $$\text{ln}\,\frac{\text{CZ}-\text{f}}{\text{f}}=\frac{{\text{nF}}_{0}\,{\text{E}}^{0}}{\text{RT}}-\frac{{\text{VnF}}_{0}}{\text{SaRT}}\,{\text{F}}_{\text{g}}$$

The logarithmic Equation (5) written in base *e* can be solved as follows:


(6) $$\frac{\text{CZ}-\text{f}}{\text{f}}={\text{e}}^{\frac{{\text{nF}}_{0}\,{\text{E}}^{0}}{\text{RT}}}\,{\text{e}}^{\left(-\frac{{\text{VnF}}_{0}}{\text{SaRT}}\,{\text{F}}_{\text{g}}\right)}$$

The impedance can be calculated as follows:


(7) $$\text{Z}=\frac{\text{f}}{\text{C}}+\frac{\text{f}}{\text{C}}\,{\text{e}}^{\frac{{\text{nF}}_{0}\,{\text{E}}^{0}}{\text{RT}}}\,{\text{e}}^{\left(-\frac{{\text{VnF}}_{0}}{\text{SaRT}}\,{\text{F}}_{\text{g}}\right)}$$

where Z is the impedance, MΩ.

In terms of a single leaf in the same situation, V, S, a, E^0^, R, T, n, F_0_, C, and f are constants. Incorporating ${\text{y}}_{0}=\frac{\text{f}}{\text{C}}$, $\text{k}=\frac{\text{f}}{\text{C}}\,{\text{e}}^{\frac{{\text{nF}}_{0}\,{\text{E}}^{0}}{\text{RT}}}$, and $\text{b}=\frac{{\text{VnF}}_{0}}{\text{SaRT}}$ into Equation (7) changes this equation to:


(8) $$\text{Z}={\text{y}}_{0}+{\text{ke}}^{-{\text{bF}}_{\text{g}}}$$

where y_0_, k, and b are the model parameters.

The derivative of Equation (8) is as follows:


(9) $${\text{Z}}^{\prime }=-{\text{bke}}^{-{\text{bF}}_{\text{g}}}$$

Leaf physiological impedance represents the resistance to current, which is generated by the transport of dielectric materials including inorganic and organic ions. LIWTR (MΩ N^–1^) is negatively correlated with the value of Z′. Therefore, LIWTR at a given F_*g*_ can be expressed as LIWTR = −Z′ = bke^−bFg^. The value of Z′ will be influenced by the external stimuli (i.e., gripping force) during the Z measurement by using the custom-made parallel-plate capacitor, −Z′at *F*_*g*_ = 0 is always calculated and used to investigate the LIWTR under natural conditions, which is defined as the inherent LIWTR (LIWTR_*i*_) of a plant, and it is calculated using Equation (9) when F_*g*_ equaled zero as follows: LIWTR_i_ = −Z′ = bk. The model parameters b and k can be estimated by fitting the relationship between Z and F_*g*_ at each treatment level, respectively.

### Measurement of Photosynthetic and Growth Indices

The net photosynthetic rate (μmol m^–2^ s^–1^), stomatal conductance (g_*s*_, mol m^–2^ s^–1^), and transpiration rate (E, mmol m^–2^ s^–1^) were measured at 9:00–11:00 a.m. with a portable LI-6400XT photosynthesis measurement system (LI-COR Inc., Lincoln, NE, United States). WUE_*i*_ (μmol mmol^–1^) was calculated according to the following equation:


(10) $${\text{WUE}}_{\text{i}}=P_{\text{N}}/E$$

The chlorophyll contents were determined by using the method described by Jawale et al. (2017). Plant height was determined by tapeline and the unit was cm, stem diameter was determined by using a Vernier caliper and the unit was mm, the measurement was conducted on the junction of root and stem, and leaf area was determined by using a leaf area meter (handheld laser leaf area meter, CI, 203) and the unit was cm^2^. The dry weights of the plants were measured at the end of the treatment. The plants were dried in an oven at 80°C. Plant dry weights of aboveground (DW_*a*_) and underground parts (DW_*u*_) were determined using an electronic analytical balance. The root/shoot ratio (R/S) was calculated as follows: R/S = DW_a_/DW_u_. The single fruit weight and fruit weight per plant were determined using an electronic analytical balance after the fruit was ripe. The economic water-use efficiency (WUE_*e*_, g L^–1^) and biomass water-use efficiency (WUE_*b*_, g L^–1^) were calculated according to the following equations:


(11) $${\text{WUE}}_{\text{e}}=\mathrm{fruit}\,\mathrm{weight}\,\mathrm{per}\,\mathrm{plant}/\mathrm{water}\,\mathrm{consumption}$$

(12) $${\text{WUE}}_{\text{b}}=\mathrm{plant}\,\mathrm{dry}\,\mathrm{weight}/\mathrm{water}\,\mathrm{consumption}$$

### Fitting Equations of the Relationship Between Growth Indices and Time

The four-parameter logistic equation (Menon and Bhandarkar, 2004) is given as follows:


(13) Y=Y+0a1+(XX0)b

where Y is the growth index, Y_0_ is the initial value during the logarithmic growth phase, a is the upper limit of the growth index, X is the number of days, X_0_ is the number of days when the growth index reaches half of the maximum value during the logarithmic growth phase, and b is a constant. GR_50_ is the growth rate at half of the logarithmic growth phase, ${\text{GR}}_{50}=\frac{-\mathrm{ab}}{4\,X_{0}}$. DT_*log*_ is the duration of the logarithmic growth phase, ${\text{DT}}_{\text{log}}=\frac{-4\,X_{0}}{\text{b}}$.

### Measurement of Nutrient Contents

Approximately 0.15–0.20 g of dried plant tissue was digested using the H_2_SO_4_-H_2_O_2_ digestion method. The N, P, and K contents were determined using the Kjeldahl, Mo-Sb antispetrophotography, and flame atomic absorption spectrophotometry methods, respectively (Xu, 2000).

### Statistical Analysis

Statistical analysis of the data was carried out using the SPSS software (version 13.0, SPSS Inc., Chicago, IL, United States) and SigmaPlot software (version 10.0, Systat Software Inc., California, CA, United States). A one-way ANOVA was used with SWC_*R*_ as the main factor. Comparisons of Ψ_*L*_, LWC, LIWTR_*i*_, chlorophyll *a* and *b* contents, N, P, and K contents, yields, WUE_*i*_, WUE_*e*_, WUE_*b*_, and R/S ratio between different treatments were conducted by the Duncan’s multiple comparison at the 5% significance level (*p* ≤ 0.05) using SPSS software. The data are shown as the means ± SE (*n* = 5). The data of *P*_*N*_ and g_*s*_ were analyzed using exploratory data analysis using SPSS software. The equations of the relationship between growth indices and time were fitted by SigmaPlot software. The Pearson correlation coefficients between LIWTR_*i*_, *P*_*N*_, g_*s*_, E, Ψ_*L*_, chlorophyll content, yields, and WUE were analyzed using SPSS software.

## Results

### Leaf Water Potential, Water Content, and Inherent Intracellular Water Transport Rate

Low SWC_*R*_ was associated with a lower Ψ_*L*_ value. The value of Ψ_*L*_ at 60% was the lowest (Table 1). The value of LWC at 100% was the highest, while that at 90% was the lowest (*p* ≤ 0.05). Since the intracellular water was part of the leaf internal retained water, the transport traits of the leaf internal retained water could be indicated by the intracellular water transport rate. LIWTR_*i*_ was the intracellular water transport rate under natural conditions, which can reflect the intrinsic leaf intracellular water status. The highest values of LIWTR_*i*_ were observed at 70 and 60%. The value of LIWTR_*i*_ at 100% was the lowest (*p* ≤ 0.05).

**TABLE 1:** Leaf water potential (Ψ_*L*_, MPa), water content (LWC, %), and inherent leaf intracellular water transport rate (LIWTR_*i*_, MΩ N^–1^) of *Solanum lycopersicum* under different water supplies.

Soil relative water content (%)	100	90	80	70	60
Ψ_*L*_ (MPa)	−0.83 a (0.019)	−0.95 b (0.011)	−1.07 c (0.041)	−1.23 d (0.011)	−1.43 e (0.029)
LWC (%)	84.55 a (0.938)	76.16 b (1.674)	82.58 ab (0.567)	80.08 ab (1.777)	79.26 ab (4.187)
LIWTR_*i*_ (MΩ N^–1^)	0.01 c (0.001)	0.02 bc (0.001)	0.04 b (0.006)	0.11 a (0.010)	0.10 a (0.011)

*Means (n = 5) in the same row followed by different letters differ significantly at p ≤ 0.05, according to one-way ANOVA (standard error is shown in parenthesis).*

### Effect of Different Water Supplies on Chlorophyll Contents and Photosynthesis

**FIGURE 1:**
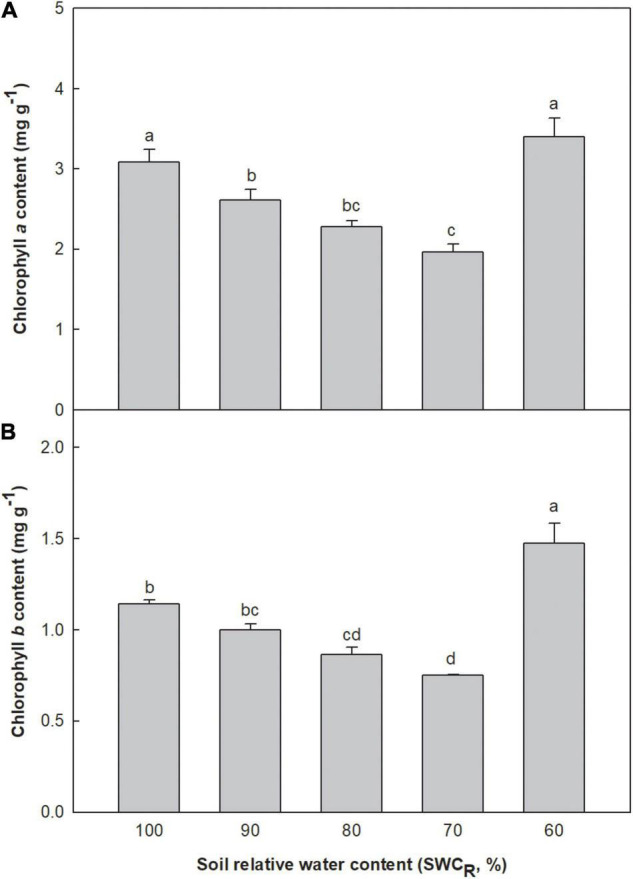
Effect of different water supplies on chlorophyll *a* and *b* contents (mg g^– 1^) [**(A)** chlorophyll *a*; **(B)** chlorophyll *b*. The means (*n* = 5) followed by different letters in the same parameter are significantly different (*p* ≤ 0.05), according to one-way ANOVA].

Low contents of chlorophyll *a* and *b* were associated with lower SWC_*R*_ between the levels ranging from 100 to 70% (Figure 1A). The content of chlorophyll *a* at 60% increased remarkably. The chlorophyll *b* content at 60% was clearly higher than that at 100% (*p* ≤ 0.05). The chlorophyll *b* contents at 70 and 80% were significantly lower than those at 100 and 90% (*p* ≤ 0.05) (Figure 1B).

**FIGURE 2:**
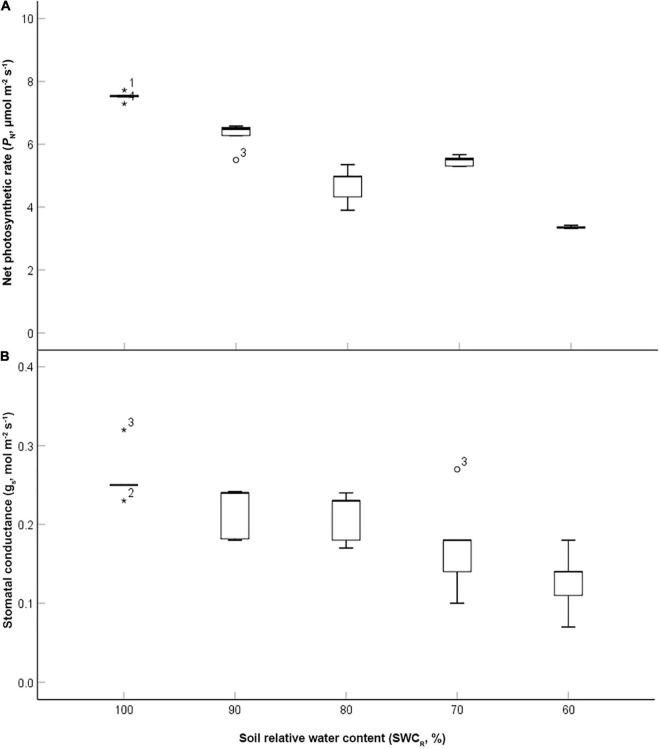
Effect of different water supplies on net photosynthetic rate (*P*_*N*_, μmol m^– 2^ s^– 1^) and stomatal conductance (g_*s*_, mol m^– 2^ s^– 1^) [**(A)** *P*_*N*_; **(B)** g_*s*_. The asterisks and circles represent the extreme outliers].

The data were analyzed using exploratory data analysis using SPSS software (version 13.0, SPSS Inc.). The value of *P*_*N*_ at 100% was the highest and that at 60% was the lowest (*p* ≤ 0.05) (Figure 2A). The low values of *P*_*N*_ were associated with lower SWC_*R*_ between the levels ranging from 100 to 80%. g_*s*_ exhibited the highest value at 100% but the lowest value at 60%. The low values of g_*s*_ were associated with lower SWC_*R*_ between the levels ranging from 80 to 60% (Figure 2B).

### Effect of Different Water Supplies on Growth Indices

**FIGURE 3:**
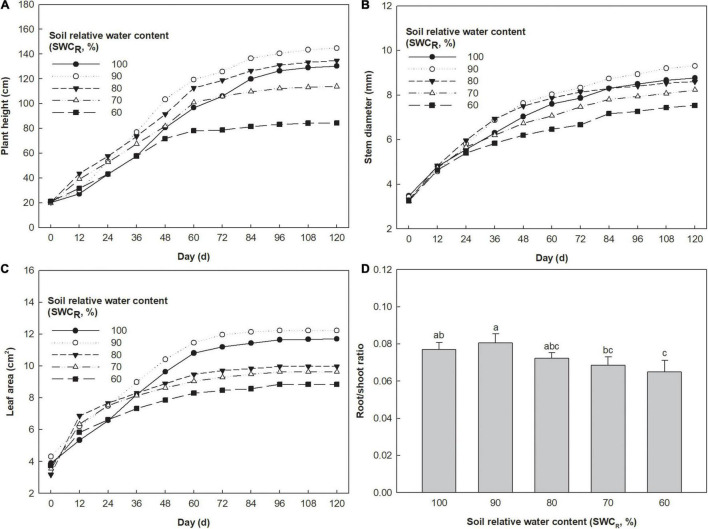
Effect of different water supplies on plant height (cm), stem diameter (mm), and leaf area (cm^2^) [**(A)** Plant height; **(B)** stem diameter; **(C)** leaf area; **(D)** root/shoot (R/S) ratio. The means (*n* = 5) followed by different letters in R/S ratio are significantly different (*p* ≤ 0.05), according to one-way ANOVA].

The plants at 90% exhibited the highest plant height, and those at 60% showed the lowest values (Figure 3A). Plant heights at 80% were higher than those at 100 and 70%. The stem diameter at 90% also showed the highest values, and the lowest values were observed at 60% (Figure 3B). The stem diameters at 100% and 80% exhibited similar growth curves, which were higher than those at 70%. The leaf areas at 90% were the highest, and those at 60% were the lowest (Figure 3C). The leaf areas at 100% were higher than those at 80 and 70%. Low R/S ratios were associated with lower SWC_*R*_ (Figure 3D). The R/S ratio at 90% was clearly higher than that at 60% (*p* ≤ 0.05).

The plant height, stem diameter, and leaf area as time increased during the treatment period were estimated by using the four-parameter logistic equation (Table 2). With respect to plant height, the values of X_0_, a, and DT_*log*_ at 100% were the highest, and those at 60% were the lowest. The values of X_0_, a, and DT_*log*_ at 80% were higher than those at 90 and 70%. The GR50 values at 90 and 70% were higher than those at 100 or 80%, and the value at 60% was the lowest. When referring to stem diameter, the values of X_0_, a, and DT_*log*_ at 80% were all higher than those at 90 or 70%. The highest values of X_0_, a, and DT_*log*_ were observed at 100%, and the lowest values were observed at 60%. The values of GR_50_ remained stable. The low values of X_0_ in leaf area were associated with lower SWC_*R*_, the values of GR_50_ in leaf area remained stable between the levels ranging from 100 to 70%, and the value at 60% was the lowest. The low values of DT_*log*_ in leaf area were associated with lower SWC_*R*_ between the levels ranging from 100 to 70%. The value of DT_*log*_ in leaf area at 60% was lower than that at 90% but higher than that at 80%.

**TABLE 2:** Fitting equations of the relationship between growth indices and time (d).

Soil relative water content (%)	Plant height (cm)				
	X_0_	a	GR_50_	DT_log_	Equation and *R*^2^
100	53.62	152.13	1.23	123.26	Y = 12.02 + $\frac{152.13}{1+{\left(\frac{X}{53.62}\right)}^{-1.74}}$ *R*^2^ = 0.99, *n* = 11, *P* < 0.0001
90	40.28	134.05	1.36	98.24	Y = 21.47 + $\frac{134.05}{1+{\left(\frac{X}{40.28}\right)}^{-1.64}}$ *R*^2^ = 0.99, *n* = 11, *P* < 0.0001
80	46.53	139.51	1.21	114.89	Y = 23.28 + $\frac{139.51}{1+{\left(\frac{X}{46.53}\right)}^{-1.62}}$ *R*^2^ = 0.99, *n* = 11, *P* < 0.0001
70	38.24	104.96	1.34	78.44	Y = 21.75 + $\frac{104.96}{1+{\left(\frac{X}{38.24}\right)}^{-1.95}}$ *R*^2^ = 0.99, *n* = 11, *P* < 0.0001
60	33.44	67.49	1.05	64.31	Y = 22.25 + $\frac{67.49}{1+{\left(\frac{X}{33.44}\right)}^{-2.08}}$ *R*^2^ = 0.99, *n* = 11, *P* < 0.0001

**Soil relative water content (%)**	**Stem diameter (mm)**				
	**X_0_**	**a**	**GR_50_**	**DT_log_**	**Equation and *R*^2^**

100	46.93	7.71	0.05	143.30	Y = 3.62 + $\frac{7.71}{1+{\left(\frac{X}{46.93}\right)}^{-1.31}}$ *R*^2^ = 0.99, *n* = 11, *P* < 0.0001
90	35.93	6.86	0.07	97.11	Y = 3.40 + $\frac{6.86}{1+{\left(\frac{X}{35.93}\right)}^{-1.48}}$ *R*^2^ = 0.99, *n* = 11, *P* < 0.0001
80	38.41	7.32	0.07	107.44	Y = 3.32 + $\frac{7.32}{1+{\left(\frac{X}{38.41}\right)}^{-1.43}}$ *R*^2^ = 0.99, *n* = 11, *P* < 0.0001
70	36.06	5.63	0.06	87.42	Y = 3.25 + $\frac{5.63}{1+{\left(\frac{X}{36.06}\right)}^{-1.65}}$ *R*^2^ = 0.99, *n* = 11, *P* < 0.0001
60	28.61	4.66	0.06	76.29	Y = 3.22 + $\frac{4.66}{1+{\left(\frac{X}{28.61}\right)}^{-1.5}}$ *R*^2^ = 0.99, *n* = 11, *P* < 0.0001

**Soil relative water content (%)**	**Leaf area (cm^2^)**				
	**X_0_**	**a**	**GR_50_**	**DT_log_**	**Fitting equations**

100	31.17	11.04	0.12	91.01	Y = 2.51 + $\frac{11.04}{1+{\left(\frac{X}{31.17}\right)}^{-1.37}}$ *R*^2^ = 0.96, *n* = 11, *P* < 0.0001
90	32.07	8.73	0.13	69.72	Y = 4.51 + $\frac{8.73}{1+{\left(\frac{X}{32.07}\right)}^{-1.84}}$ *R*^2^ = 0.99, *n* = 11, *P* < 0.0001
80	26.96	6.15	0.11	55.30	Y = 4.26 + $\frac{6.15}{1+{\left(\frac{X}{26.96}\right)}^{-1.95}}$ *R*^2^ = 0.99, *n* = 11, *P* < 0.0001
70	21.4	6.50	0.13	51.26	Y = 3.58 + $\frac{6.5}{1+{\left(\frac{X}{21.4}\right)}^{-1.67}}$ *R*^2^ = 0.99, *n* = 11, *P* < 0.0001
60	19.88	5.61	0.09	60.24	Y = 3.73 + $\frac{5.61}{1+{\left(\frac{X}{19.88}\right)}^{-1.32}}$ *R*^2^ = 0.99, *n* = 11, *P* < 0.0001

### Effect of Different Water Supplies on N, P, and K Contents in Plant Tissues

The N and P contents in leaves, stems, and roots all decreased with decreasing SWC_*R*_ (*p* ≤ 0.05) (Table 3). The N contents in leaves, stems, and roots at 60% were 80.25, 79.78, and 79.32% of those at 100%, respectively. The P contents in leaves, stems, and roots at 60% were only 68.09, 66.27, and 65.90% of those at 100%, respectively. The N contents in leaves at each water supply level were the highest, while those in roots were the lowest (*p* ≤ 0.05). The P contents in leaves at each water supply level were also the highest, while those in stems were the lowest (*p* ≤ 0.05). The lowest K content in leaves was observed at 80%, and the highest value was observed at 100% (*p* ≤ 0.05). Low K contents in stems and roots were associated with lower SWC_*R*_. The K contents in stems and roots at 60% were only 89.39% and 81.54% of those at 100%, respectively.

**TABLE 3:** Effect of different water supplies on N, P, and K contents in plant leaves, stems, and roots.

Soil relative water content (%)	N (g kg^–1^)			P (g kg^–1^)			K (g kg^–1^)		
	Leaves	Stems	Roots	Leaves	Stems	Roots	Leaves	Stems	Roots
100	7.14 a (0.04)	6.33 a (0.03)	6.14 a (0.03)	1.88 a (0.02)	1.69 a (0.01)	1.73 a (0.01)	6.64 a (0.04)	6.60 a (0.02)	4.93 a (0.03)
90	6.84 b (0.02)	6.00 b (0.03)	5.79 b (0.04)	1.85 a (0.02)	1.65 a (0.01)	1.70 a (0.01)	6.53 b (0.01)	6.54 ab (0.03)	4.86 b (0.01)
80	6.77 b (0.01)	5.91 c (0.02)	5.71 c (0.02)	1.73 b (0.01)	1.51 b (0.02)	1.57 b (0.02)	6.28 d (0.02)	6.48 b (0.03)	4.66 c (0.02)
70	6.31 c (0.02)	5.53 d (0.01)	5.26 d (0.02)	1.53 c (0.01)	1.30 c (0.01)	1.35 c (0.01)	6.33 d (0.03)	6.20 c (0.01)	4.34 d (0.01)
60	5.73 d (0.02)	5.05 e (0.03)	4.87 e (0.02)	1.28 d (0.02)	1.12 d (0.03)	1.14 d (0.03)	6.43 c (0.01)	5.90 d (0.02)	4.02 e (0.02)

*Means (n = 5) in the same column followed by different letters differ significantly at p ≤ 0.05, according to one-way ANOVA (standard error is shown in parenthesis).*

### Effect of Different Water Supplies on Yields of Tomatoes

The water consumption per plant, leaf dry weight, plant dry weight, single fruit weight, fruit weight per plant, and yield increase at 90% were the highest (Table 4). The everyday average water supply volumes (ml) at each water treatment level were shown in Supplementary Table 1. In addition, the change of soil water potential as time increased was shown in Supplementary Figure 1, which was previously determined by our research team. Leaf dry weight decreased with decreasing SWC_*R*_ between the levels ranging from 90 to 60%. The plant dry weight clearly decreased and became only 45.50 g when SWC_*R*_ decreased to 70% (*p* ≤ 0.05). The low values of single fruit weight or fruit weight per plant were associated with lower SWC_*R*_ between the levels ranging from 80 to 60% (*p* ≤ 0.05). The water consumption per plant at 80% was clearly lower than that at 100% (*p* ≤ 0.05). The plants at 60% exhibited the lowest water consumption, plant dry weight, single fruit weight, fruit weight per plant, and yield increase (*p* ≤ 0.05). Production declines were observed in the 70 and 60% SWC_*R*_ treatments.

**TABLE 4:** Effect of different water supplies on yields of tomatoes.

Soil relative water content (%)	Water consumption per plant (L)	Leaf dry weight (g)	Plant dry weight (g)	Single fruit weight (g)	Fruit weight per plant (g)	Yields increase (%)
100	26.84 b (0.13)	11.67 b (0.93)	50.67 ab (2.20)	53.96 b (1.79)	215.83 bc (7.17)	—
90	29.05 a (0.52)	18.50 a (1.32)	56.33 a (2.77)	60.88 a (0.26)	243.50 a (1.04)	12.82
80	23.98 c (0.10)	10.00 bc (0.50)	51.67 a (1.64)	55.79 b (0.87)	223.17 b (3.49)	3.40
70	19.06 d (0.21)	8.67 c (0.60)	45.50 b (1.15)	41.40 c (1.03)	207.00 c (5.13)	−4.09
60	12.49 e (0.26)	3.67 d (0.44)	36.50 c (0.87)	33.80 d (0.55)	169.00 d (2.75)	−21.70

*Means (n = 5) in the same column followed by different letters differ significantly at p ≤ 0.05, according to one-way ANOVA (standard error is shown in parenthesis).*

### Effect of Different Water Supplies on Water-Use Efficiency

The high values of WUE_*i*_, WUE_*e*_, and WUE_*b*_ were associated with lower SWC_*R*_ (Table 5). The values of WUE_*i*_ and WUE_*b*_ clearly increased at 70%, while a significant increase in the value of WUE_*e*_ was observed at 80%. The highest values of WUE_*i*_, WUE_*e*_, and WUE_*b*_ were all observed at 60% (*p* ≤ 0.05).

**TABLE 5:** Instant water-use efficiency (WUE_*i*_, μmol mmol^–1^), economic water-use efficiency (WUE_*e*_, g L^–1^), and biomass water-use efficiency (WUE_*b*_, g L^–1^) in plants subjected to different water supplies.

Soil relative water content (%)	100	90	80	70	60
WUE_*i*_ (μmol mmol^–1^)	1.41 c (0.17)	1.44 c (0.13)	1.73 bc (0.14)	2.09 ab (0.05)	2.33 a (0.11)
WUE_*e*_ (g L^–1^)	8.04 d (0.23)	8.39 d (0.13)	9.31 c (0.10)	10.86 b (0.20)	13.54 a (0.47)
WUE_*b*_ (g L^–1^)	1.89 c (0.09)	1.94 c (0.12)	2.16 bc (0.08)	2.39 b (0.05)	2.93 a (0.13)

*Means (n = 5) in the same row followed by different letters differ significantly at p ≤ 0.05, according to one-way ANOVA (standard error is shown in parenthesis).*

### Relationship Between LIWTR_*i*_, P_*N*_, g_*s*_, E, Ψ_*L*_, Chlorophyll Content, Yields, and WUE_*i*_

The Pearson correlation coefficients for the relationship between LIWTR_*i*_, *P*_*N*_, g_*s*_, E, Ψ_*L*_, chlorophyll content, yields, and WUE_*i*_ are shown in Table 6. LIWTR_*i*_ exhibited a good correlation with Ψ_*L*_, *P*_*N*_, g_*s*_, E, yields, and WUE_*i*_. Ψ_*L*_ also showed a good correlation with the abovementioned parameters. LIWTR_*i*_ could represent plant water status and Ψ_*L*_. Yields also showed a significant relationship with WUE_*i*_. However, chlorophyll content exhibited no significant relationship with other parameters. In addition, LIWTR_*i*_ has not yet been directly linked to the photosynthesis and growth in this study.

**TABLE 6:** The Pearson correlation coefficients among leaf intracellular water transport rate (LIWTR_*i*_, MΩ N^–1^), net photosynthetic rate (*P*_*N*_, μmol m^–2^ s^–1^), stomatal conductance (g_*s*_, mmol m^–2^ s^–1^), transpiration rate (E, mmol m^–2^ s^–1^), leaf water potential (Ψ_*L*_, MPa), chlorophyll content (chl, mg^–1^ g^–1^), yields (g), and instant water-use efficiency (WUE_*i*_, μmol mmol^–1^) (*n* = 25).

	*P*_*N*_	g_*s*_	E	Ψ_*L*_	Chl	Yields	WUE_*i*_
LIWTR_*i*_	−0.690**	−0.747**	−0.815**	−0.904**	−0.347	−0.702**	0.803**
*P*_*N*_		0.900**	0.884**	0.835**	0.220	0.585*	−0.617*
g_*s*_			0.868**	0.875**	0.188	0.609*	−0.740**
E				0.937**	0.330	0.623*	−0.878**
Ψ_*L*_					0.189	0.773**	−0.894**
Chl						−0.096	−0.215
Yields							−0.726**

*Correlation is significant at the 0.05 level (2-tailed).

**Correlation is significant at the 0.01 level (2-tailed).

## Discussion

### Leaf Water Status Under Different Water Supplies

The variation in physiological impedance is correlated with the cytosolic solute concentration in leaves, and it represents the intracellular water or dielectric material transport traits (Garcia-Navarro et al., 2019). The LIWTR was calculated by determining the variation rate of the physiological impedance at increasing gripping forces (Xing et al., 2021). Decreasing Ψ_*L*_ might be caused by the increased cytosolic solute concentration (Yamani et al., 2020). Decreased Ψ_*L*_ improved the water absorption capacity of leaves at 90% compared with that at 100% (Table 1). A higher growth rate of roots rather than the aboveground part was also conducive to water absorption in plants at 90% (Figure 3D). As a result, LIWTR_*i*_ remained stable, and intracellular water could be constantly delivered and utilized in leaves at 90%. Sustained exposure to water deficit often results in increased leaf respiration, probably in response to an increase in energy demand as leaves cope with water stress (Sanhueza et al., 2015). Decreasing water supply at 80% limited the water uptake by the plant, but the transpiration dissipation kept stable as a result of the maintenance of g_*s*_, which caused a further decrease in Ψ_*L*_. Ψ_*L*_ governs water transport between cells (Taiz et al., 2015). However, metabolic water produced during respiration or other biochemistry processes at 80% together with the decreasing water consumption by *P*_*N*_ would maintain or even slightly increase the LWC. Meanwhile, the improved LIWTR_*i*_ at 80% was conducive to the efficient use of leaf internal retained water.

Carbohydrates accumulated during the photosynthetic process in plants not only participate in the promotion of plant growth but also play important roles in many important physiological processes, such as resistance to adversity (Xing et al., 2019). The water produced during carbohydrate consumption helped to maintain the stability of the LWC and g_*s*_ at 70%. However, the remarkable increase in LIWTR_*i*_ indicated that the transport of intracellular water or nutrients was clearly improved at 70%. As a result, limited intracellular water and mineral elements could be efficiently delivered and utilized by the photosynthetic apparatus. The uptake of water by roots was inhibited by the decreasing water supply at 60%. However, decreases in Ψ_*L*_, g_*s*_, *P*_*N*_, growth, and dry matter accumulation at 60% reduced the water consumption and maintained the LWC compared with that at 70% (Figure 2). The transport rate of the leaf intracellular water at 60% remained the same as that at 70%, which was conducive to other biochemistry reactions, i.e., respiration or chlorophyll biosynthesis. Energy or metabolic water produced during respiration helped plants cope with water deficit.

### N, P, and K Contents, Photosynthesis, and Plant Growth

With a 100% SWC_*R*_, the plant roots would be in hypoxia preventing their growth and development. In this study, the root growth (Figure 3D) of tomatoes at 100% exhibited a slight but not significant decrease compared with that at 90%, which would also cause an effect on the water uptake by roots. We also found that the tomato plants at 100% consumed less water than those at 90%. The changes in photosynthesis and growth might be attributed to the leaf intracellular water transport traits.

Stomatal closure happening at 90% SWC_*R*_ decreased the *P*_*N*_ of tomato. The synthesis of leaf chlorophyll *a* content was inhibited at 90%, which would also reduce *P*_*N*_ (Figure 1A). However, the water carbon exchange efficiency in plants at 90% remained stable, which was indicated by WUE_*i*_. Since the intracellular nutrients could be constantly delivered and utilized in leaves, plants at 90% increased the growth rates of plant height, stem diameter, and leaf area to improve the accumulation of dry matter, which increased the leaf and plant dry weight, fruit weight, and yield but decreased the water content in leaves. Therefore, although g_*s*_ of plants decreased in a timely manner to reduce transpiration when SWC_*R*_ decreased to 90%, the water consumption per plant at 90% still increased significantly, and WUE_*e*_ and WUE_*b*_ were not improved at 90%.

The decreased leaf area reduced the transpiration consumption per plant at 80% compared with that at 90%, therefore maintaining g_*s*_ (Figure 3C). The *P*_*N*_ of tomatoes at 80% was inhibited due to the decreased nutrient contents, including N, P, and K in plants, and the decreased N, P, and K uptake by plants might be attributed to the decreasing water availability in soil. However, plants had consistent water carbon exchange efficiency at 80% compared with that at 90%. Although the decreased *P*_*N*_ slowed the growth rate of plant height, the plants at 80% could still extend the duration of the logarithmic growth phase of plant height and stem diameter. Respiration would consume carbohydrates in plants at 80%, which caused a decline in leaf dry weight and yield. However, the yields at 80% still increased by 3.40% compared to that in the control, and WUE_*e*_ exhibited an increase at 80% (Table 4). In fact, the increase in LIWTR_*i*_ improved the transport of intracellular water, which was mainly supplied for plant growth (Table 1).

The N and P contents in all plant tissues and the K contents in stems and roots clearly decreased at 70%, which inhibited the synthesis of chlorophyll *a* and *b* in leaves (Figure 1). Plant growth, including plant height, stem diameter, and leaf area, was also decreased (Figure 3). However, the efficient delivery of the intracellular water and mineral elements was conducive to maintain *P*_*N*_ of tomatoes at 70% compared with that at 80% (Figure 2). Consequently, WUE_*i*_, WUE_*e*_, and WUE_*b*_ were all improved (Table 5). Although the photosynthetic capacity remained stable compared to that at 80%, the portion of photosynthetic accumulated organic matter that was used for promoting plant growth declined, and plants at 70% needed greater carbohydrate consumption to adapt to the adversity, which would also cause a decline in dry matter accumulation and yields. Decreased nutrient uptake by roots caused by the decreasing water supply significantly inhibited the *P*_*N*_ at 60%. In addition, the single fruit weight at 60% became only 62.64% of that in the control. Besides, the remarkable increase in the content of chlorophyll *a* and *b* was mainly attributed to the clearly decreased leaf dry weight and stable chlorophyll biosynthesis at 60% (Shi et al., 2010).

**FIGURE 4:**
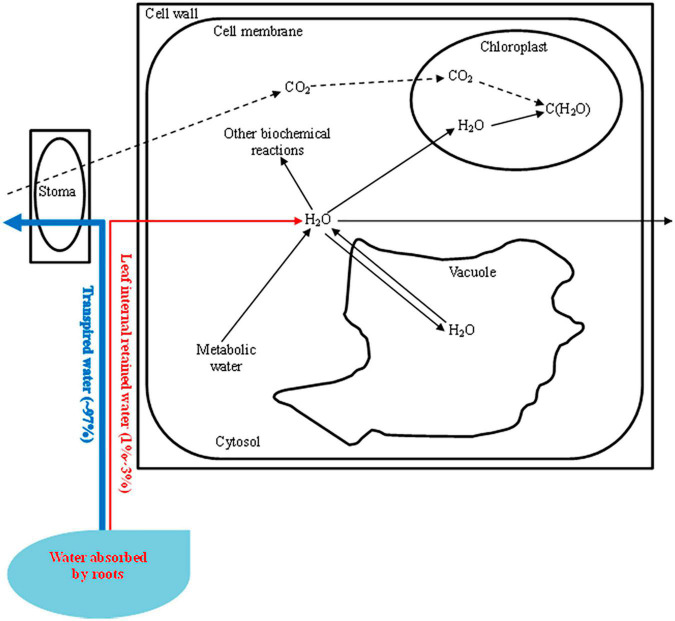
Leaf intracellular water transport [most (∼97%) of the water absorbed by roots is transported through the stoma. Leaf internal retained water (1–3%) is transported in cells and is supplied to the photosynthetic apparatus and other biochemical reactions. The intracellular water status can also be regulated by metabolic water. The leaf water potential (Ψ_*L*_) can be influenced by the solute concentration in the vacuole, while the inherent leaf intracellular water transport rate (LIWTR_*i*_) is related to the transport of intracellular water or dielectric materials. Decreasing the water supply will increase the LIWTR_*i*_ but decrease the Ψ_*L*_, and the variation in LIWTR_*i*_ combined with Ψ_*L*_ determines the utilization traits of the intracellular water].

In this research, we also observed a good correlation between the LIWTR_*i*_ and *P*_*N*_, g_*s*_, Ψ_*L*_, yields, and WUE_*i*_. The utilization of the intracellular water was determined by the intracellular water transport traits and Ψ_*L*_ (Figure 4), which played important roles in the photosynthesis, dry matter and yield accumulation, and efficient use of the leaf internal retained water. However, the intracellular water status can also be influenced by the leaf anatomy and mechanical strength, and the investigation of the synergism of these two indices and LIWTR on leaf internal retained water status helps to improve the accuracy of the determination of plant water requirement information, which will improve the WUE of tomatoes and promote the application of electrophysiological indices in water determination. Furthermore, the LIWTR in this research is not directly linked to the regulation of photosynthesis and growth, and the establishment of the direct relationship between leaf internal retained water and photosynthesis and growth needs further research.

## Conclusion

Changes in leaf intracellular water transport traits and Ψ_*L*_ altered the availability of the leaf intracellular water, which played an important role in photosynthesis, dry matter and yield accumulation, and the efficient use of intracellular water. When SWC_*R*_ was 100, 90, and 80%, stomatal closure reduced the transpiration and decreased the water transport within leaves as SWC_*R*_ decreased, and *P*_*N*_ was inhibited by the decreasing g_*s*_ or Ψ_*L*_, but the constant transport of intracellular water was conducive to plant growth or dry matter accumulation. A remarkable increase in LIWTR_*i*_ helped to improve the delivery and WUE of the retained leaf internal water and nutrients, which maintained the *P*_*N*_ and improved the WUE at 70% but could not keep the plant growth and yields of tomatoes at 70 and 60% due to the further decrease of water supply and Ψ_*L*_. The results demonstrate the importance of the transport of the leaf intracellular water in plant responses to water deficit by using electrophysiological parameters and provide a basis for further research on the water requirement information in plants and for the improvement of WUE.

## Data Availability Statement

The original contributions presented in the study are included in the article/Supplementary Material, further inquiries can be directed to the corresponding author.

## Author Contributions

DX and YW designed the research and conducted the analyses. YW conceived and funded the research. DX and RM wrote the manuscript. ZL designed and conducted the experiments in greenhouse. XQ analyzed the data of physiological impedance. WF revised the manuscript. All authors contributed to the article and approved the submitted version.

## Conflict of Interest

The authors declare that the research was conducted in the absence of any commercial or financial relationships that could be construed as a potential conflict of interest.

## Publisher’s Note

All claims expressed in this article are solely those of the authors and do not necessarily represent those of their affiliated organizations, or those of the publisher, the editors and the reviewers. Any product that may be evaluated in this article, or claim that may be made by its manufacturer, is not guaranteed or endorsed by the publisher.

## Supplementary Material

Supplementary Figure 1 The soil water potential (MPa) as time (Day, d) increased. The soil used in this experiment is clay, CKc represents the soil water content accounts for 85–90% of the field water capacity of clay, Tc1 represents the soil water content accounts for 70–75% of the field water capacity, Tc2 represents the soil water content accounts for 60–65% of the field water capacity, Tc3 represents the soil water content accounts for 50–55% of the field water capacity. The water supply was stopped from the second day.
