# Supplementary figures and images for: Leaf Intracellular Water Transport Rate Based on Physiological Impedance: A Possible Role of Leaf Internal Retained Water in Photosynthesis and Growth of Tomatoes
Source: Front Plant Sci. 2022 Apr 1;13:845628. doi: 10.3389/fpls.2022.845628 (PMC9010976; doi:10.3389/fpls.2022.845628)

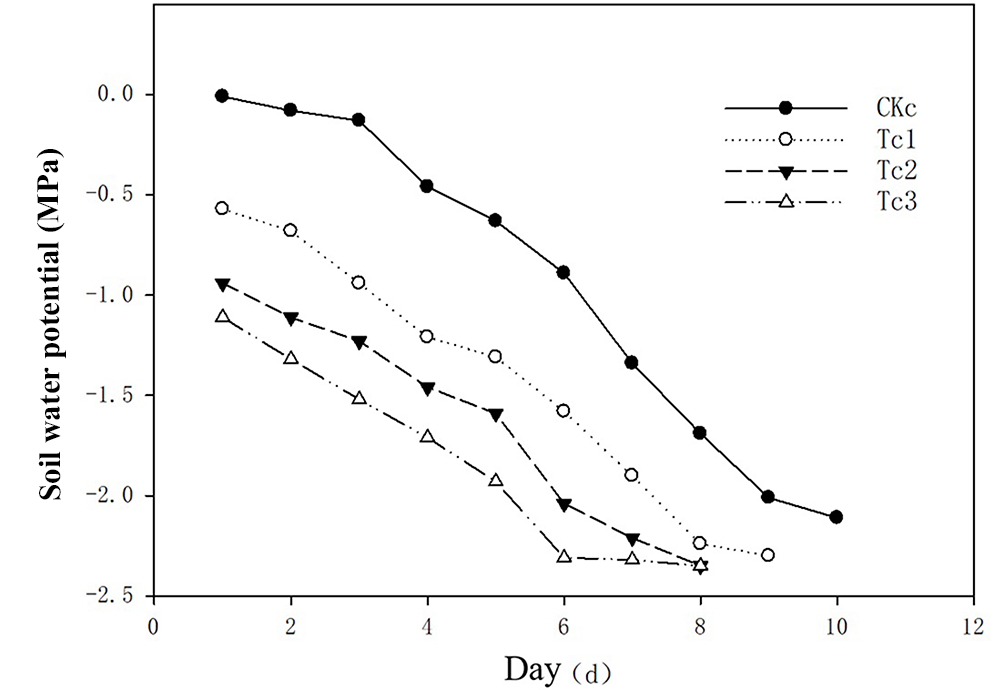

Supplement: Supplementary Figure 1 — The soil water potential (MPa) as time (Day, d) increased. The soil used in this experiment is clay, CKc represents the soil water content accounts for 85–90% of the field water capacity of clay, Tc1 represents the soil water content accounts for 70–75% of the field water capacity, Tc2 represents the soil water content accounts for 60–65% of the field water capacity, Tc3 represents the soil water content accounts for 50–55% of the field water capacity. The water supply was stopped from the second day. [file Image_1.tif]
