# Supplementary material for: Leaf Intracellular Water Transport Rate Based on Physiological Impedance: A Possible Role of Leaf Internal Retained Water in Photosynthesis and Growth of Tomatoes
Source: Front Plant Sci. 2022 Apr 1;13:845628. doi: 10.3389/fpls.2022.845628 (PMC9010976; doi:10.3389/fpls.2022.845628)
Supplement: Supplementary file 2 [file Table_1.DOCX]

Supplementary Material

## Supplementary Tables

**Supplementary Table 1.** The everyday average water supply volumes (mL) at each water treatment level.

| Date | 100% | 90% | 80% | 70% | 60% |
| --- | --- | --- | --- | --- | --- |
| 2019.09.01 | 2089 | 1819 | 1566 | 1328 | 1104 |
| 2019.09.02 | 216 | 143 | 141 | 93 | 82 |
| 2019.09.03 | 269 | 203 | 161 | 135 | 119 |
| 2019.09.04 | 337 | 276 | 235 | 215 | 185 |
| 2019.09.05 | 290 | 198 | 185 | 179 | 175 |
| 2019.09.06 | 282 | 296 | 233 | 188 | 150 |
| 2019.09.07 | 387 | 244 | 182 | 172 | 176 |
| 2019.09.08 | 304 | 290 | 218 | 201 | 196 |
| 2019.09.09 | 290 | 308 | 264 | 222 | 198 |
| 2019.09.10 | 296 | 310 | 278 | 218 | 185 |
| 2019.09.11 | 269 | 270 | 225 | 177 | 149 |
| 2019.09.12 | 211 | 282 | 211 | 212 | 240 |
| 2019.09.13 | 361 | 378 | 336 | 313 | 261 |
| 2019.09.14 | 321 | 353 | 303 | 262 | 218 |
| 2019.09.15 | 276 | 270 | 228 | 179 | 147 |
| 2019.09.16 | 278 | 259 | 230 | 202 | 156 |
| 2019.09.17 | 269 | 231 | 253 | 224 | 176 |
| 2019.09.18 | 305 | 355 | 307 | 286 | 200 |
| 2019.09.19 | 283 | 235 | 226 | 224 | 130 |
| 2019.09.20 | 296 | 250 | 270 | 259 | 142 |
| 2019.09.21 | 210 | 212 | 240 | 237 | 149 |
| 2019.09.22 | 210 | 220 | 202 | 246 | 133 |
| 2019.09.23 | 204 | 263 | 251 | 234 | 163 |
| 2019.09.24 | 209 | 272 | 260 | 237 | 161 |
| 2019.09.25 | 293 | 253 | 260 | 256 | 153 |
| 2019.09.26 | 246 | 224 | 284 | 223 | 143 |
| 2019.09.27 | 228 | 217 | 268 | 239 | 129 |
| 2019.09.28 | 304 | 350 | 283 | 239 | 158 |
| 2019.09.29 | 274 | 309 | 244 | 173 | 113 |
| 2019.09.30 | 268 | 297 | 247 | 105 | 151 |
| 2019.10.01 | 254 | 289 | 236 | 196 | 150 |
| 2019.10.02 | 272 | 304 | 241 | 179 | 94 |
| 2019.10.03 | 235 | 272 | 202 | 100 | 96 |
| 2019.10.04 | 287 | 209 | 197 | 108 | 94 |
| 2019.10.05 | 236 | 269 | 194 | 128 | 96 |
| 2019.10.06 | 275 | 303 | 246 | 176 | 87 |
| 2019.10.07 | 302 | 347 | 280 | 218 | 128 |
| 2019.10.08 | 256 | 295 | 139 | 196 | 99 |
| 2019.10.09 | 245 | 280 | 114 | 178 | 93 |
| 2019.10.10 | 212 | 241 | 195 | 164 | 98 |
| 2019.10.11 | 239 | 285 | 205 | 162 | 84 |
| 2019.10.12 | 276 | 310 | 247 | 185 | 107 |
| 2019.10.13 | 334 | 379 | 300 | 240 | 142 |
| 2019.10.14 | 236 | 270 | 219 | 165 | 137 |
| 2019.10.15 | 194 | 223 | 173 | 116 | 51 |
| 2019.10.16 | 247 | 283 | 235 | 176 | 95 |
| 2019.10.17 | 207 | 250 | 196 | 131 | 92 |
| 2019.10.18 | 225 | 263 | 215 | 158 | 96 |
| 2019.10.19 | 276 | 312 | 263 | 185 | 105 |
| 2019.10.20 | 185 | 215 | 174 | 126 | 67 |
| 2019.10.21 | 144 | 283 | 229 | 186 | 45 |
| 2019.10.22 | 212 | 252 | 203 | 139 | 76 |
| 2019.10.23 | 286 | 323 | 277 | 200 | 115 |
| 2019.10.24 | 214 | 254 | 200 | 145 | 90 |
| 2019.10.25 | 156 | 296 | 239 | 187 | 45 |
| 2019.10.26 | 135 | 161 | 221 | 182 | 40 |
| 2019.10.27 | 224 | 263 | 211 | 163 | 90 |
| 2019.10.28 | 250 | 280 | 229 | 173 | 90 |
| 2019.10.29 | 234 | 272 | 222 | 164 | 94 |
| 2019.10.30 | 188 | 282 | 195 | 134 | 74 |
| 2019.10.31 | 158 | 158 | 238 | 127 | 62 |
| 2019.11.01 | 152 | 176 | 154 | 98 | 47 |
| 2019.11.02 | 244 | 266 | 231 | 156 | 86 |
| 2019.11.03 | 253 | 292 | 258 | 167 | 92 |
| 2019.11.04 | 220 | 248 | 214 | 156 | 74 |
| 2019.11.05 | 175 | 186 | 178 | 112 | 68 |
| 2019.11.06 | 268 | 288 | 241 | 173 | 94 |
| 2019.11.07 | 215 | 238 | 200 | 129 | 67 |
| 2019.11.08 | 168 | 253 | 162 | 144 | 91 |
| 2019.11.09 | 147 | 198 | 132 | 116 | 74 |
| 2019.11.10 | 183 | 190 | 178 | 157 | 93 |
| 2019.11.11 | 209 | 231 | 186 | 158 | 89 |
| 2019.11.12 | 173 | 190 | 156 | 140 | 82 |
| 2019.11.13 | 185 | 196 | 152 | 139 | 76 |
| 2019.11.14 | 190 | 212 | 183 | 138 | 84 |
| 2019.11.15 | 212 | 239 | 203 | 141 | 85 |
| 2019.11.16 | 242 | 266 | 220 | 164 | 93 |
| 2019.11.17 | 189 | 205 | 180 | 134 | 79 |
| 2019.11.18 | 265 | 290 | 232 | 184 | 138 |
| 2019.11.19 | 269 | 292 | 228 | 187 | 141 |
| 2019.11.20 | 173 | 204 | 155 | 111 | 70 |
| 2019.11.21 | 161 | 195 | 226 | 100 | 63 |
| 2019.11.22 | 233 | 253 | 202 | 162 | 96 |
| 2019.11.23 | 239 | 273 | 213 | 186 | 128 |
| 2019.11.24 | 122 | 157 | 100 | 65 | 38 |
| 2019.11.25 | 83 | 115 | 71 | 48 | 25 |
| 2019.11.26 | 154 | 190 | 138 | 95 | 53 |
| 2019.11.27 | 220 | 252 | 195 | 140 | 94 |
| 2019.11.28 | 65 | 89 | 64 | 72 | 32 |
| 2019.11.29 | 118 | 131 | 107 | 74 | 39 |
| 2019.11.30 | 142 | 159 | 118 | 87 | 43 |
| 2019.12.01 | 85 | 104 | 74 | 45 | 28 |
| 2019.12.02 | 127 | 146 | 104 | 69 | 38 |
| 2019.12.03 | 76 | 92 | 68 | 49 | 32 |
| 2019.12.04 | 122 | 143 | 104 | 75 | 44 |
| 2019.12.05 | 126 | 143 | 116 | 83 | 49 |
| 2019.12.06 | 94 | 106 | 88 | 61 | 37 |
| 2019.12.07 | 78 | 86 | 71 | 46 | 34 |
| 2019.12.08 | 125 | 144 | 115 | 80 | 55 |
| 2019.12.09 | 73 | 82 | 64 | 43 | 29 |
| 2019.12.10 | 70 | 78 | 62 | 45 | 28 |
| 2019.12.11 | 152 | 97 | 81 | 74 | 99 |
| 2019.12.12 | 144 | 143 | 120 | 100 | 45 |
| 2019.12.13 | 124 | 465 | 109 | 88 | 37 |
| 2019.12.14 | 110 | 114 | 93 | 87 | 42 |
| 2019.12.15 | 125 | 131 | 106 | 85 | 36 |
| 2019.12.16 | 124 | 140 | 101 | 86 | 46 |
| 2019.12.17 | 130 | 146 | 114 | 75 | 42 |
| 2019.12.18 | 110 | 115 | 100 | 80 | 36 |
| 2019.12.19 | 113 | 92 | 97 | 77 | 36 |
| 2019.12.20 | 123 | 115 | 102 | 81 | 41 |
| 2019.12.21 | 119 | 128 | 99 | 80 | 41 |
| 2019.12.22 | 70 | 75 | 64 | 46 | 31 |
| 2019.12.23 | 105 | 123 | 95 | 68 | 34 |
| 2019.12.24 | 60 | 71 | 57 | 45 | 30 |
| 2019.12.25 | 45 | 47 | 38 | 26 | 14 |
| 2019.12.26 | 47 | 54 | 32 | 39 | 16 |
| 2019.12.27 | 120 | 157 | 123 | 89 | 45 |
| 2019.12.28 | 116 | 130 | 86 | 74 | 43 |
| 2019.12.29 | 45 | 53 | 41 | 26 | 19 |
| 2019.12.30 | 57 | 62 | 53 | 41 | 23 |
| 2019.12.31 | 91 | 98 | 85 | 68 | 40 |
| 2020.01.01 | 106 | 112 | 99 | 74 | 41 |
| 2020.01.02 | 39 | 42 | 45 | 33 | 27 |
| 2020.01.03 | 26 | 32 | 10 | 8 | 5 |
| 2020.01.04 | 45 | 49 | 40 | 29 | 16 |
| 2020.01.05 | 95 | 107 | 90 | 72 | 47 |
| 2020.01.06 | 112 | 125 | 101 | 80 | 54 |
| 2020.01.07 | 63 | 71 | 58 | 41 | 23 |
| 2020.01.08 | 103 | 116 | 82 | 62 | 31 |
| 2020.01.09 | 54 | 63 | 48 | 32 | 18 |
| 2020.01.10 | 106 | 114 | 96 | 67 | 36 |
| 2020.01.11 | 49 | 54 | 43 | 30 | 15 |
| Total | 26842 | 29049 | 23977 | 19057 | 12491 |
